# Rotor Attitude Estimation for Spherical Motors Using Geometry-Constrained Kalman Transformer Algorithm in Monocular Vision

**DOI:** 10.3390/s26103156

**Published:** 2026-05-16

**Authors:** Fucong Liu, Baokaidi Tian, Faqiang Wen, Lei Yu, Tianxiang Yu, Min Li

**Affiliations:** 1 School of Mechanical Engineering, Tianjin University of Technology and Education, Tianjin 300222, China; 2 Tianjin Intelligent Robot Technology and Application Enterprise Key Laboratory, Tianjin 300222, China; 3 Tianjin BestBond (Baishibingde) Intelligent Technology Co., Ltd., Tianjin 300300, China

**Keywords:** permanent magnet spherical motor, rotor attitude estimation, visual tracking, Kalman filter, transformer

## Abstract

Permanent-magnet spherical motors (PMSpMs) possess three-degree-of-freedom omnidirectional motion characteristics, and rotor attitude estimation (RAE) is essential for closed-loop control. This article proposes a visual RAE method for spherical motors using a Kalman filter and geometric constraint Transformer (GK-TransT). An RAE system was equipped with a monocular area scan camera with a visual feature component (VFC) mounted on the bottom of the rotor. In the proposed GK-TransT algorithm, the Kalman filter is used to enhance the robustness and accuracy of the TransT tracker. To verify the algorithm, a tracking comparison was conducted among the GK-TransT, original TransT, KCF, and CSRT algorithms. The results indicate that the tracking precisions of the proposed GK-TransT algorithm for the main and auxiliary feature points reach 90.9% and 94.4%, respectively, with an average processing speed of 61.23 FPS and a single-frame latency of 16.33 ms. Considering the tracking precision, real-time performance, and robustness under occlusion and motion blur conditions, the GK-TransT algorithm is more applicable for the RAE of the PMSpM. In addition, an RAE test bench was developed, and the GK-TransT-based method and a micro-electro-mechanical system (MEMS) sensor were compared. The physical ground truth of a hydraulic rotary table was used as the benchmark. The comparison results indicate that the GK-TransT-based method achieves a higher accuracy than the MEMS method. Finally, the practicability of the proposed method is proved.

## 1. Introduction

Spherical motors are novel actuators that can realize 3-DOF omnidirectional motion independently [1,2,3]. They can be effectively used to replace the traditional complex transmission systems composed of multiple single-DOF motors connected in series and parallel. Due to their compact structure and lack of kinematic singularity, spherical motors have broad application prospects in fields such as humanoid robot joints, spacecraft attitude control, and industrial gimbals. However, rotor attitude estimation (RAE) using spherical motors is much more difficult than with traditional single-axis rotating motors due to complex spatial coupling motion characteristics. Currently, detection schemes capable of satisfying the requirements of highly dynamic closed-loop control are still required, and the accurate acquisition of rotor attitude remains a key challenge in this field. After years of research, existing RAE methods can be mainly divided into two major categories: contact and non-contact types.

Contact-type detection methods primarily rely on direct physical contact between mechanical encoders and the rotor to resolve the attitude information [4,5]. However, the omnidirectional motion range of spherical motors is restricted by the integration of external measurement devices. More critically, the mechanical friction induced by the contact not only results in additional output torque loss but also further deteriorates the long-term accuracy of RAE due to operational wear.

Non-contact detection has gradually emerged as the mainstream research direction in this field. Existing non-contact measurement schemes can be primarily classified into three categories: electromagnetic, micro-electro-mechanical system (MEMS), and optical-based approaches.

In electromagnetic schemes, Hall sensor arrays or compressed sensing techniques are primarily utilized to resolve the rotor position [2,6,7,8]. However, in practical applications, these sensors are easily affected by the internal non-linear magnetic fields of the motor and the alternating magnetic fields of the coils. Consequently, such methods inevitably encounter electromagnetic coupling interference.

In MEMS-based approaches, low-cost MEMS inertial sensors are primarily utilized to acquire attitude data [9,10]. Benefiting from the high sampling rate and relatively mature resolution system, MEMS sensors have become a commonly adopted method for evaluating the attitude algorithms of spherical motors. However, they also suffer from inherent bias drift. Furthermore, the mechanical inertia of the rotor increases due to the introduction of a wireless power transmission (WPT) module at the rotor side, thereby limiting its long-term operational stability. To circumvent electromagnetic interference and the additional physical payload on the rotor, external measurement methods based on monocular vision have received widespread attention in recent years. An improved fast discriminative scale space tracking (FDSST) algorithm was introduced by Xue et al. [11] to achieve the localization and attitude resolution of the rotor. Subsequently, kinematic models and visual tracking were further combined by Zhou et al. [12,13], and visual RAE systems based on the multi-object Kalman KCF and KMFDSST algorithms were successively proposed, effectively alleviating the tracking failure problem caused by external short-term occlusion during rotor operation.

Despite the progress achieved in the aforementioned studies, the underlying architectures of current visual RAE schemes are primarily based on traditional correlation filters. In such algorithms, target templates are mainly constructed by relying on shallow, hand-crafted features. When encountering complex conditions, such as high-speed motion blur induced by the sudden, rapid motion of the motor, severe perspective distortion caused by large-angle rotations, and target occlusion, the shallow templates are highly susceptible to contamination, thereby triggering irreversible tracking drift. Consequently, significant limitations still exist in the robustness and recapturing capability of the system. To address these issues, a visual tracking algorithm based on the Transformer attention mechanism (TransT) has been applied to the permanent-magnet spherical motor (PMSpM) detection. Furthermore, this article presents an improved visual RAE method integrating Kalman temporal prediction and rigid body geometric constraints (GK-TransT).

The main contributions of this article are summarized as follows:

1. A monocular vision method for the RAE of PMSpM is proposed. By introducing TransT into the visual RAE, the proposed method improves tracking robustness under high-speed motion blur and short-term target occlusion conditions compared to traditional correlation filter-based tracking methods.
2. The GK-TransT algorithm is developed by fusing Kalman temporal prediction and a rigid body geometric prior. By combining primary feature point tracking with auxiliary feature point localization via geometric constraints, this method avoids the parallel tracking of dual feature points using independent deep models, thereby reducing single-frame processing latency and improving the real-time performance of visual feedback.
3. An RAE test bench using a high-precision hydraulic rotary table as the physical benchmark is constructed. Experimental results show that the proposed visual method provides higher accuracy in both static and dynamic attitude measurements compared with the conventional MEMS sensor scheme, and it is not affected by the cumulative bias drift of the MEMS.

## 2. PMSpM Structure and Mathematical Model of Rotor Attitude

### 2.1. PMSpM Structure and Coordinate System

**Figure 1:**
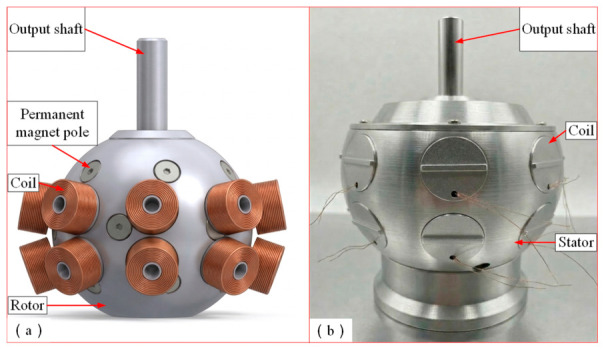
Basic structure of the PMSpM: (**a**) 3D model of the PMSpM; (**b**) prototype of the PMSpM.

**Figure 2:**
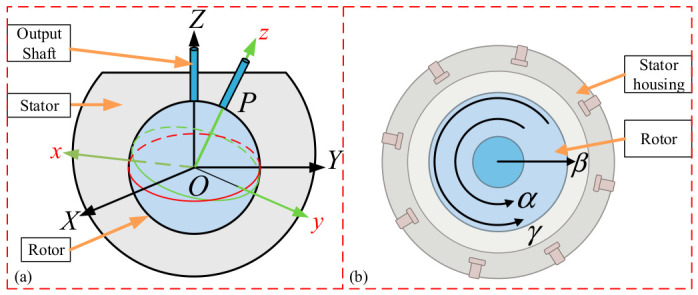
Coordinate systems of PMSpM: (**a**) stator and rotor coordinates; (**b**) Z-Y-Z Euler angles.

In this article, a PMSpM [14] is adopted as the research object, and its structure is illustrated in Figure 1. The stator of the adopted PMSpM is configured with a total of 16 winding coils at two layers, while 18 permanent magnets (PMs) are distributed on the rotor surface according to an icosahedral topological structure (with the two spatial pole positions removed), whereby the spatial 3-DOF rotational motion can be realized. The stator coordinate system (O-XYZ) and rotor coordinate system (o-xyz) are defined, where the origins of both systems are fixed at the motor center O and are coincident in the initial state. The spatial attitude of the rotor relative to the stator is represented by a standard Z-Y-Z Euler angle sequence, which describes the state after the rotor coordinate system is successively rotated about the stator’s Z-axis by the spin angle α, the Y-axis by the tilt angle β, and the Z-axis again by the yaw angle γ, as shown in Figure 2.

### 2.2. Overview of the Mathematical Model

**Figure 3:**
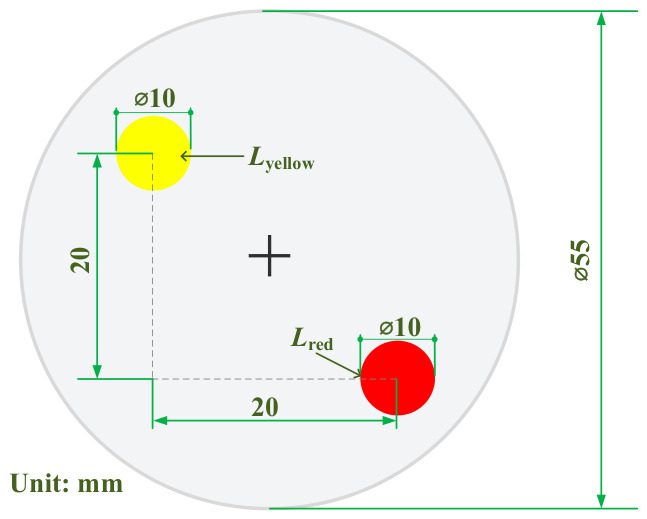
Geometric configuration of the VFC.

**Figure 4:**
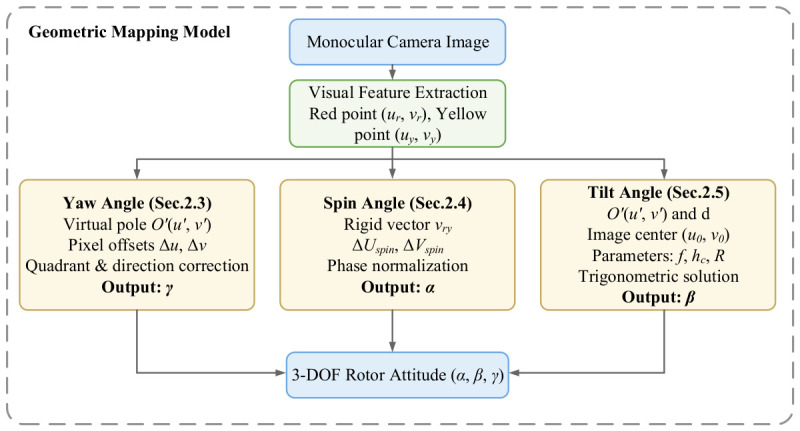
Architecture of the geometric mapping model.

A non-contact detection scheme utilizing a bottom-mounted, upward-viewing monocular area scan camera is adopted. To acquire the rotor attitude, a visual feature component (VFC) is rigidly mounted at the bottom pole position of the rotor as a measurement target. The geometric configuration of the VFC is illustrated in Figure 3. Two tracking feature points, the main and auxiliary points, are established on the VFC. After the pixel coordinates of the dual feature points and their midpoint (defined as the virtual pole) are extracted, the 3-DOF attitude angles of the rotor can be directly resolved through a geometric mapping model in combination with the aforementioned Z-Y-Z Euler angle spatial reference, as illustrated by the overall architecture in Figure 4.

### 2.3. Calculation of Yaw Angle

The resolution of the yaw angle relies on the virtual rotation center $O^{\prime }$ comprising the main and auxiliary tracking points. Based on the rigid body geometric symmetry of the target surface, the pixel coordinates $\left(u^{\prime },v^{\prime }\right)$ of the virtual center $O^{\prime }$ can be derived from the midpoint between the main feature point $\left(u_{r},v_{r}\right)$ and the auxiliary feature point $\left(u_{y},v_{y}\right)$ as follows:

(1) $$u^{\prime }=\frac{u_{r}+u_{y}}{2},\;v^{\prime }=\frac{v_{r}+v_{y}}{2}.$$

**Figure 5:**
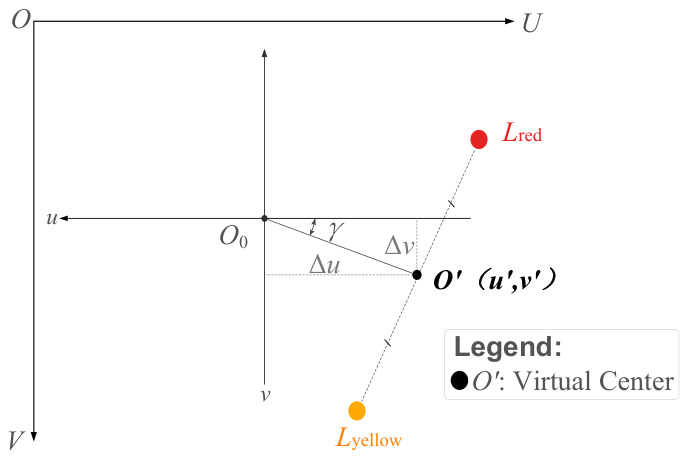
Schematic of yaw angle calculation.

As illustrated in Figure 5, based on the relative geometric relationship between the virtual center $O^{\prime }$ and the image center O in the pixel plane, the temporary yaw angle can be readily obtained as

(2) $$\gamma_{temp}=\arctan\left(\left|\frac{\Delta v}{\Delta u}\right|\right),$$

where $\gamma_{temp}$ denotes the temporary yaw angle without considering the quadrant status, and Δu and Δv represent the components of the virtual center $O^{\prime }$ along the U-axis and V-axis in the pixel coordinate system, respectively. Based on the spatial configuration of the bottom-mounted, upward-viewing monocular area scan camera, specific geometric projection relationships exist in the visual imaging model of the system. Therefore, corresponding coordinate compensation is required during the attitude resolution.

Primarily, an inverse projection relationship exists in the translation direction. When the physical tilt of the rotor occurs, since the feature points are rigidly mounted at the bottom of the rotor, their displacement direction in the image plane is opposite to the actual tilt direction of the rotor. To align the pixel displacement vector with the direction of the stator coordinate system, a sign inversion process must be applied to the extracted pixel offsets, $\Delta u=-\left(u^{\prime }-u_{0}\right)$, $\Delta v=-\left(v^{\prime }-v_{0}\right)$, where $\left(u_{0},v_{0}\right)$ denotes the coordinates of the image center O.

Since the temporary yaw angle $\gamma_{temp}$ is an acute angle, whereas the actual yaw angle ranges from 0 to 2π, the calculated yaw angle $\gamma_{calc}$ can be derived by considering the quadrant relationship between Δu and Δv, as expressed in the following equation:

(3) $$\gamma_{calc}=\left\{\begin{array}{ll} \gamma_{temp} & \left(\Delta u>0,\Delta v>0\right)\\ \pi -\gamma_{temp} & \left(\Delta u<0,\Delta v>0\right)\\ \pi +\gamma_{temp} & \left(\Delta u<0,\Delta v<0\right)\\ 2\pi -\gamma_{temp} & \left(\Delta u>0,\Delta v<0\right) \end{array}\right..$$

Furthermore, a mirror mapping relationship exists regarding the rotation direction. Under the upward-viewing perspective, a counterclockwise rotation of the rotor in physical space manifests as a clockwise rotation when projected onto the 2-D image plane. Therefore, subsequent to quadrant mapping, rotation direction correction and phase normalization must be applied to the calculated yaw angle to guarantee the continuity of the angle resolution as follows:

(4) $$\gamma_{final}=\left\{\begin{array}{ll} 2\pi -\gamma_{calc} & \left(\gamma_{calc}\neq 0\right)\\ 0 & \left(\gamma_{calc}=0\right) \end{array}\right..$$

### 2.4. Calculation of Spin Angle

Theoretically, when physical tilt occurs in the rotor, the projection of the rotational trajectory of the bottom feature points onto the camera imaging plane is distorted from a standard circle into an ellipse. If the arctangent function is directly utilized within the 2-D pixel plane to resolve the spin angle, a certain projection error will be introduced. However, for the investigated PMSpM, the mechanical tilt angle of the rotor is restricted to a relatively minor range (0°~15°) by its structural design.

Considering that the adopted monocular area scan camera features a prolonged working distance (where the object distance $h_{c}$ is significantly larger than the rotor radius R) and a narrow field of view, the non-linear impact on pixel coordinates resulting from the foreshortening effect induced by the minute tilt angle is limited. To balance the accuracy and computational efficiency of the attitude resolution, this higher-order projection distortion is neglected under small-tilt conditions, and an approximately decoupled 2-D geometric projection model is applied to compute the spin angle.

Based on the aforementioned approximate model, the resolution of the spin angle necessitates two visual feature points, i.e., $L_{\mathrm{red}}$ and $L_{\mathrm{yellow}}$, which are conceptualized as a rigid body directional vector, denoted as $v_{ry}$. The azimuth angle of this vector directly characterizes the real-time spin attitude of the rotor. The horizontal and vertical components of the feature vector $v_{ry}$ within the image pixel coordinate system (*O*-*UV*) are defined as $\Delta U_{spin}=u_{y}-u_{r}$ and $\Delta V_{spin}=v_{y}-v_{r}$, respectively.

In a standard image coordinate system, the positive direction of the V-axis points vertically downward by default. Since the industrial camera is mounted at the bottom of the PMSpM for upward-viewing observation, the counterclockwise rotational motion within the physical space of the stator manifests as a clockwise rotation when mapped onto the image plane (i.e., deflecting from the +U-axis toward the +V-axis). This mirror effect, induced by the upward-viewing perspective, precisely compensates for the geometric characteristics of the downward-pointing V-axis in the image coordinate system.

Consequently, no additional coordinate axis inversion transformation is required during the attitude resolution process. By directly introducing the two-variable arctangent function to resolve the azimuth angle of the feature vector within the image coordinate system, the initial value of the true physical spin angle $\alpha_{0}$ conforming to the right-hand rule (with counterclockwise defined as the positive direction) can be acquired. To ensure the continuity of the angular values and uniformly map them to the absolute interval of [0,2π), a piecewise phase normalization is applied as follows:

(5) $$\alpha_{0}=\arctan(\Delta V_{\mathrm{spin}},\Delta U_{\mathrm{spin}}),$$

(6) $$\alpha =\left\{\begin{array}{ll} \alpha_{0}, & \alpha_{0}\geq 0\\ \alpha_{0}+2\pi , & \alpha_{0}<0 \end{array}\right..$$

**Figure 6:**
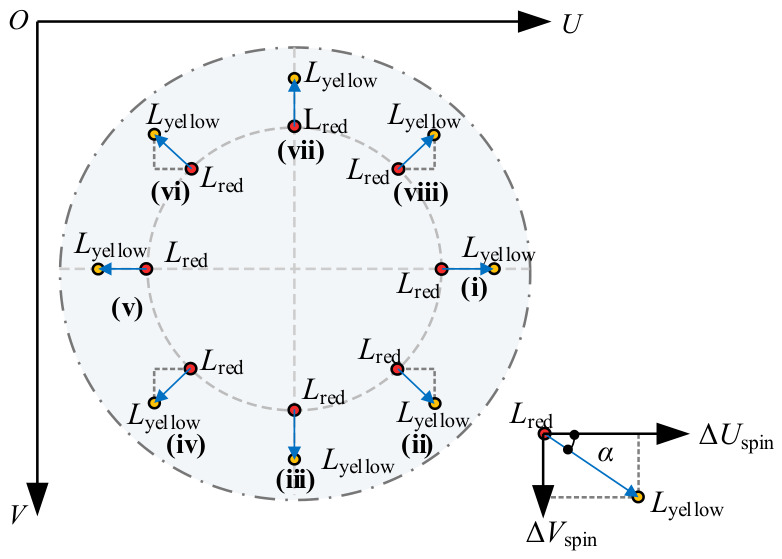
Schematic of spin calculation.

Figure 6 explicitly illustrates the spin angle mapping relationships under eight distinct quadrants and boundary conditions based on this principle, thereby further verifying the continuity and robustness of the resolution method throughout the entire motion cycle.

### 2.5. Calculation of Tilt Angle

The adopted monocular area scan camera satisfies the pinhole camera model. To eliminate the coupling effect of the rotor’s spin motion on the tilt angle calculation, the tilt angle must be independently resolved based on the geometric center of the visual feature group at the bottom of the rotor.

In this visual model, since the main feature point $L_{\mathrm{red}}$ and the auxiliary feature point $L_{\mathrm{yellow}}$ are strictly symmetrically distributed on both sides of the central pole at the rotor bottom, the midpoint of their connection line on the image plane physically corresponds strictly to the actual bottom pole of the rotor. Assuming the coordinates of these two visual feature points within the image coordinate system are $\left(u_{r},v_{r}\right)$ and $\left(u_{y},v_{y}\right)$, respectively, the coordinates of the geometric center (i.e., the virtual center $O^{\prime }$) of this feature group can be expressed as

(7) $$u^{\prime }=\frac{u_{r}+u_{y}}{2},\;v^{\prime }=\frac{v_{r}+v_{y}}{2}.$$

**Figure 7:**
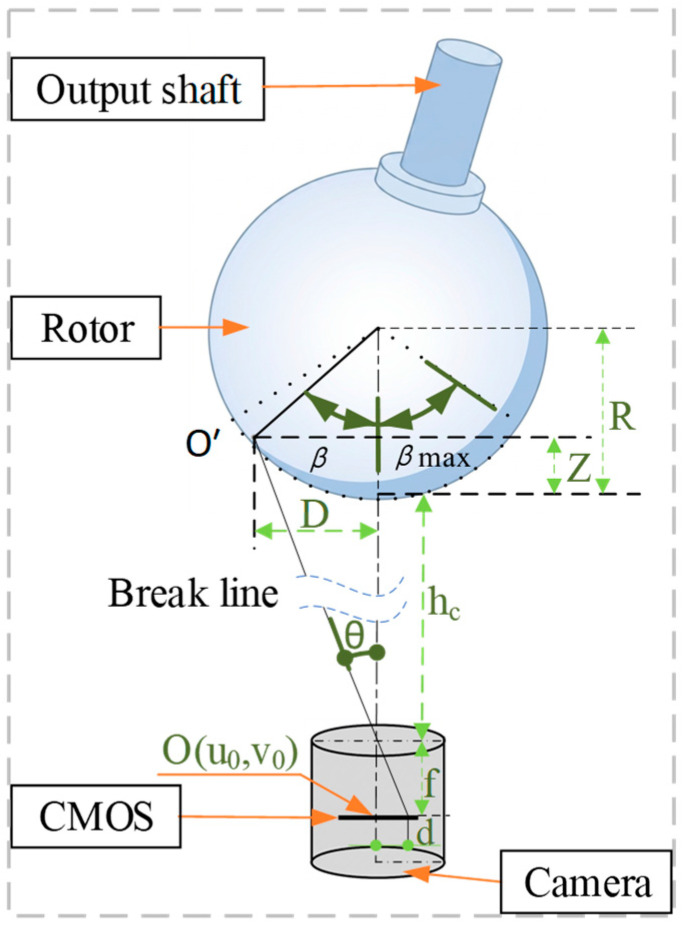
Schematic of tilt calculation.

Initially, the original attitude of the rotor is defined such that the output shaft direction aligns with the direction of the stator coordinate system. Figure 7 illustrates the spatial geometric relationship between the monocular area scan camera and the spherical motion range of the geometric center of the visual feature group when the rotor deflects from its initial attitude by a specific angle β, where β denotes the tilt angle relative to the stator coordinate system.

Based on Figure 7, the following relationships can be derived:

(8) $$\frac{D}{d}=\frac{h_{c}+\Delta Z}{f}\;,$$

where D denotes the projection distance of the geometric center of the visual feature group at the rotor bottom from its current position to the initial position along the XOY plane; ΔZ represents the displacement of this geometric center along the Z-axis direction; d denotes the physical displacement norm of this geometric center on the image sensor plane relative to the image center $\left(u_{0},v_{0}\right)$; f is the focal length of the monocular area scan camera; and $h_{c}$ represents the vertical distance from the optical center of the camera to the initial plane of the geometric center at the rotor bottom. Consequently, it can be deduced that

(9) D=Rsinβ;

(10) ΔZ=R(1−cosβ); and

(11) $$d=\sqrt{{\left[(u^{\prime }-u_{0})du\right]}^{2}+{\left[(v^{\prime }-v_{0})dv\right]}^{2}}.$$

Herein, R denotes the radius of the spherical motion trajectory of the geometric center of the visual feature group at the rotor bottom. Substituting the aforementioned equations into Equation (8), the following relationship can be derived:

(12) $$\frac{R\sin\beta }{h_{c}+R(1-\cos\beta )}=\frac{d}{f}.$$

Let an intermediate variable be defined as $C=\frac{d}{f}$, where d denotes the image displacement of the geometric center and f represents the focal length. Based on the geometric relationship, the following trigonometric equation can be derived:

(13) $$\sin\left(\beta +\arctan(C)\right)=\frac{C\left(h_{c}+R\right)}{R\sqrt{1+C^{2}}},$$

where the coefficient term satisfies $\frac{C}{\sqrt{1+C^{2}}}=\frac{d}{\sqrt{f^{2}+d^{2}}}$. By simplifying the right side of the aforementioned equation and substituting the result, the final analytical calculation formula for the rotor tilt angle β can be obtained as

(14) $$\beta =\arcsin\left(\frac{(h_{c}+R)\cdot d}{R\sqrt{f^{2}+d^{2}}}\right)-\arctan\left(\frac{d}{f}\right).$$

## 3. The Proposed GK-TransT Algorithm for Tracking and Detection

### 3.1. Backbone Visual Tracking Network

**Figure 8:**
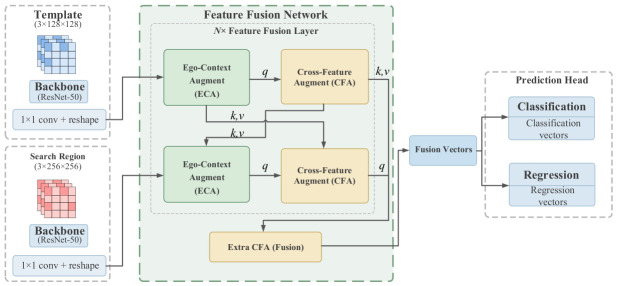
Network architecture of the original TransT algorithm.

Traditional tracking algorithms based on Siamese networks are limited by the local linear matching of cross-correlation, rendering them susceptible to interference from similar backgrounds under complex working conditions. To achieve highly robust tracking of the rotor feature points, the TransT algorithm based on the attention mechanism is adopted in this system as the foundational visual backbone network [15]. Its overall structure is illustrated in Figure 8.

The image features of the target template (z) and the search region (x) are extracted in parallel by this network using an improved ResNet-50. To establish long-range global contextual dependencies, Ego-Context Augment (ECA) and Cross-Feature Augment (CFA) modules were introduced into the core of the network. Their underlying feature interaction is based on the Scaled Dot-Product Attention mechanism, which is mathematically expressed as

(15) $$Attention(Q,K,V)=\mathrm{softmax}\left(\frac{QK^{T}}{\sqrt{d_{k}}}\right)V\;,$$

where Q, K, and V represent the query, key, and value matrices, respectively; $d_{k}$ is the feature dimension. The internal structural representation of the features is extracted by the ECA module through the self-attention mechanism, whereas the CFA module utilizes the cross-attention mechanism to achieve the deep fusion of the template and search region features. The fused feature sequence is input into the Multi-Layer Perceptron (MLP) prediction head for the classification and regression of the target bounding box. During the training phase of the base network, its jointly optimized target loss function $L_{\mathrm{total}}$ is defined as

(16) $$L_{\mathrm{total}}=\lambda_{cls}L_{cls}+\lambda_{reg}L_{reg}+\lambda_{gious}L_{gious},$$

where $\lambda_{cls}$, $\lambda_{reg}$, and $\lambda_{giou}$ respectively represent the balanced weight coefficients of each loss term.

### 3.2. The Proposed GK-TransT Algorithm

During RAE, target loss and tracking drift are highly likely to occur when image sequences are compromised by environmental interferences, such as object occlusion, illumination variations, or sample contamination. Furthermore, traditional multi-landmark localization necessitates the parallel instantiation of multiple independent TransT models. The multiplied computational overhead consequently leads to severe single-frame processing latency, which struggles to satisfy the real-time requirements of closed-loop control.

To address these challenges, this article proposes an improved tracking algorithm integrating the GK-TransT. Based on a hybrid architecture of “single-model guidance combined with lightweight geometric resolution,” a spatio-temporal joint compensation mechanism is constructed by this algorithm. In the temporal dimension, the Kalman filter is introduced for state prediction to compensate for visual observations under low confidence. In the spatial dimension, combined with rigid body geometric constraints, a local region of interest (ROI) is established using the main feature point as the benchmark to extract the auxiliary feature point. Subsequently, spatial distance verification is conducted utilizing the relative error between the pixel distance and the physical prior. If the auxiliary point fails verification or the extraction is unsuccessful, the relative position vector from the previous valid frame is directly invoked by the system for coordinate reconstruction. This architecture circumvents the parallel computation of multiple models. Consequently, while the tracking robustness under complex conditions is guaranteed, the overall execution speed of the algorithm is significantly enhanced.

#### 3.2.1. Occlusion-Resistant Prediction Mechanism via Kalman Filter

Under operating conditions characterized by high-speed rotor rotation or landmark occlusion, the prediction head of the TransT algorithm is susceptible to outputting low-confidence bounding boxes, which may even lead to tracking drift. To address the aforementioned issue, a Kalman filter is introduced into the tracking framework of the main feature point [16,17].

The discrete state-space model of the target tracking system is formulated by the following state transition equation and observation equation:

(17) $$X_{k}=FX_{k-1}+W_{k-1},$$

(18) $$Z_{k}=HX_{k}+V_{k},$$

where $W_{k-1}$ and $V_{k}$ represent the process noise and measurement noise, respectively, which are assumed to be independent zero-mean Gaussian white noise processes. In this implementation, the Kalman filter parameters were fixed for all test sequences. The process noise covariance matrix Q and measurement noise covariance matrix R were set as $Q=0.01I_{4}$ and $R=0.1I_{2}$, respectively, where $I_{4}$ and $I_{2}$ denote the 4×4 and 2×2 identity matrices. The relatively small process noise was adopted to maintain smooth prediction during short-term occlusion, while the measurement noise was selected to allow reliable visual observations to correct the predicted state without introducing excessive frame-level jitter. These values were empirically tuned according to the coordinate fluctuation of the visual feature points and the response smoothness under dynamic tracking conditions.

Let the state vector be defined as the two-dimensional position and velocity of the target within the image coordinate system, denoted as $X_{k}={\left[x,y,v_{x},v_{y}\right]}^{T}$. Assuming the target undergoes uniform linear motion between adjacent frames and the sampling time interval is set as a unit frame Δt, the discrete state transition matrix F can be formulated as

(19) $$F=\left[\begin{array}{cccc} 1 & 0 & 1 & 0\\ 0 & 1 & 0 & 1\\ 0 & 0 & 1 & 0\\ 0 & 0 & 0 & 1 \end{array}\right].$$

The discrete observation vector $Z_{k}={\left[x_{vis},y_{vis}\right]}^{T}.$ is directly constituted by the center coordinates of the bounding box output by the TransT prediction head. The corresponding observation matrix H is defined as

(20) $$H=\left[\begin{array}{cccc} 1 & 0 & 0 & 0\\ 0 & 1 & 0 & 0 \end{array}\right].$$

During the frame-by-frame iteration of the algorithm, a visual observation confidence evaluation mechanism is constructed based on the HSV color space. Specifically, feature validation is conducted by calculating the proportion of valid non-zero pixels within the ROI extracted by TransT. The HSV threshold ranges for the red marker are set as $H_{r}\in [0,10]\cup [170,180]$, $S_{r}\in [50,255]$, and $V_{r}\in [50,255]$, while those for the yellow marker are set as $H_{y}\in [15,40]$, $S_{y}\in [50,255]$, and $V_{y}\in [50,255]$, following the OpenCV HSV convention. These specific ranges are determined based on the statistical sampling of the markers’ color distributions under the experimental illumination conditions. When this valid pixel proportion exceeds a predefined empirical threshold of 0.05, the current visual observation is determined to be valid, and the output value of TransT is utilized to perform the Kalman state update. This 0.05 threshold was empirically selected based on partial occlusion tests to retain valid marker observations while rejecting severely occluded or contaminated observations.

Conversely, when the valid pixel proportion is lower than this threshold, the current visual observation is regarded as unreliable, and the predicted coordinates from the Kalman filter are used as the main feature point position for the current frame.

#### 3.2.2. Cooperative Feature Localization Based on Rigid Body Geometric Constraints

The rotor of the PMSpM can be regarded as a standard rigid body, and the visual feature points rigidly attached to its surface maintain a strict geometric positional relationship in three-dimensional space. In the designed VFC, the main feature point (red target) and the auxiliary feature point (yellow target) are arranged diagonally, with their physical right-angle side distances both being 20 mm. Therefore, theoretically, the absolute physical distance between the two feature points is a constant, L = 28.28 mm.

Based on this physical prior constraint, a cooperative localization mechanism is designed for the auxiliary feature point:

1. Spatial Distance Verification: Utilizing the main feature point as the primary anchor, the coordinates of auxiliary candidate points are extracted within a predefined dynamic radius region via HSV color space threshold segmentation and morphological processing. Combined with the pixel scale S (pixels/mm) of the current frame, the actual pixel distance $D_{det}$ between the candidate point and the primary anchor is calculated. Considering the foreshortening effect induced by the rotor tilt, the tilt angle β resolved in the previous frame is introduced for dynamic projection compensation, and the expected pixel distance $D_{exp}$ is calculated as
  (21) $$D_{exp}=LS\cos(\beta ),$$
  where β denotes the tilt angle resolved in the previous frame. Subsequently, the relative error proportion E is calculated as
  (22) $$E=\frac{\left|D_{det}-D_{exp}\right|}{D_{exp}}.$$
  The candidate point is determined to be valid when E<0.3. This 30% geometric error tolerance is set as a conservative threshold to account for the projection approximation error within the rotor tilt range of 0°~15°, the inter-frame attitude update lag, and the uncertainties caused by motion blur and pixel-level feature extraction. Such a setting reduces the possibility of falsely rejecting valid auxiliary feature points under dynamic tilt and local blur conditions while suppressing pseudo-feature interferences caused by environmental reflections or background noise.
2. Position Compensation Under Feature Absence: When environmental interferences (such as physical occlusion, abrupt illumination changes, or feature sample contamination) occur in the image sequence, causing the auxiliary feature point to be invisible or fail the aforementioned error verification, the relative pixel vector $V_{\mathrm{rel}}$ between the main and auxiliary feature points preserved in the previous high-confidence frame is extracted. Based on the reliable coordinates $P_{red}$ of the current main feature point, the auxiliary feature point coordinates $P_{yellow}$ are directly estimated for compensation via spatial geometric projection:
  (23) $$P_{yellow}=P_{red}+{\vec{V}}_{rel}.$$
  The aforementioned cooperative localization mechanism reconstructs traditional dual-target tracking into a hybrid architecture of “single-model guidance combined with lightweight geometric resolution.” While ensuring tracking robustness under complex operating conditions, the computational overhead of instantiating an independent deep learning model for the auxiliary feature point is circumvented, thereby satisfying the real-time requirements of closed-loop control.

#### 3.2.3. Execution Workflow of the Algorithm

After the coordinates of the main and auxiliary feature points are acquired via frame-by-frame iteration, provided that both points remain in a valid observation state, the Exponential Moving Average (EMA) algorithm is introduced to smoothly update the pixel scale $S_{t}$. This is implemented to suppress the high-frequency jitter induced by single-frame visual extraction

(24) $$S_{t}=0.9S_{t-1}+0.1\left(\frac{D_{det}}{L_{physical}}\right),$$

where $S_{t-1}$ denotes the pixel scale of the previous frame; $D_{det}$ represents the detected pixel distance between the two points in the current frame; and L is the true physical distance between the two feature points, which is 28.28 mm. To prevent scale distortion induced by large-angle perspective distortion, the aforementioned update equation is executed exclusively within the steady-state interval where the rotor operates at a minor tilt angle (e.g., β<2°). Under large-tilt maneuvering conditions, $S_{t}$ is kept locked to avoid the introduction of cumulative computational errors.

**Figure 9:**
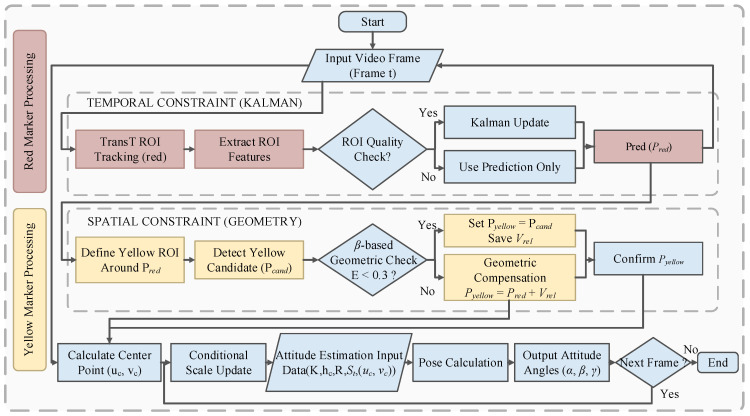
Flowchart of the GK-TransT algorithm.

Based on the aforementioned spatio-temporal joint mechanism, the midpoint of the line connecting the main and auxiliary feature points is extracted as the virtual pole $\left(u_{c},\;v_{c}\right)$ of the rotor rotation, which serves as the final observation input. Combined with the pre-calibrated camera intrinsic matrix K and the physical radius R of the rotor, the 3-DOF spatial attitude of the rotor is inversely resolved. The complete execution workflow of the GK-TransT algorithm is illustrated in Figure 9.

## 4. Validation of the GK-TransT Algorithm

### 4.1. Construction and Annotation of the Offline Visual Dataset

**Figure 10:**
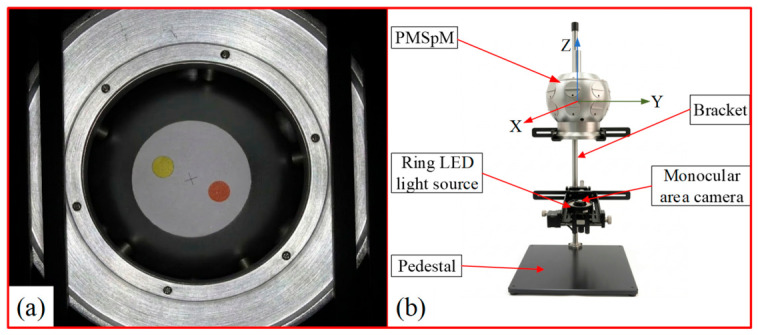
Rotor recognition and video dataset acquisition device for the PMSpM: (**a**) Prototype of the VFC. (**b**) Video data acquisition setup.

To verify the accuracy and robustness of the proposed GK-TransT algorithm in target tracking tasks, a dedicated video data acquisition and testing platform for the PMSpM was established. The prototype of the VFC and the video data acquisition setup are illustrated in Figure 10a and Figure 10b, respectively.

During the experimental data acquisition phase, the motion of the PMSpM rotor was manually manipulated, and a monocular area scan camera was utilized to record its continuous motion trajectory. Subsequently, the acquired video sequences were annotated frame by frame using the CVAT image annotation tool, thereby constructing an offline visual dataset suitable for validating the PMSpM RAE algorithms.

The offline comparison and evaluation of all algorithms were conducted on an ASUS mobile workstation. The hardware configuration of this workstation comprised an Intel Core i9-13980HX CPU, 32 GB of RAM, and an NVIDIA GeForce RTX 4060 GPU. The software verification environment was based on Python 3.8.

### 4.2. Tracking Accuracy Comparison of Algorithms

**Figure 11:**
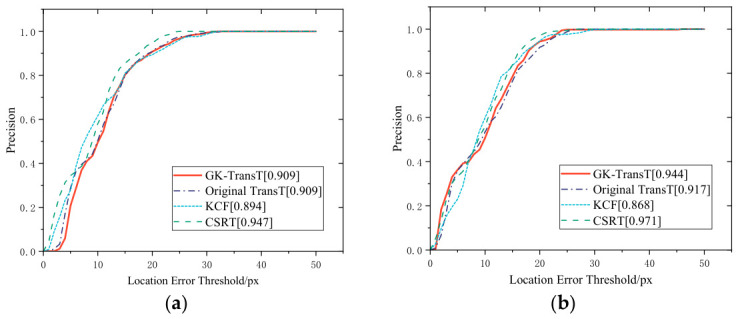
Comparison of tracking algorithm accuracy: (**a**) Tracking accuracy of the main feature point. (**b**) Tracking accuracy of the auxiliary feature point.

To verify the comprehensive tracking performance of the algorithms, a comparative analysis was conducted among the GK-TransT, original TransT, CSRT, and KCF algorithms based on the aforementioned offline visual dataset. The precision curves of each algorithm for the main and auxiliary feature points are illustrated in Figure 11.

According to Figure 11, when the center location error threshold is set to 20 px, the tracking precisions of the CSRT algorithm for the primary and secondary markers are 94.7% and 97.1%, respectively. The precisions of the proposed GK-TransT algorithm for the primary and secondary markers are 90.9% and 94.4%, respectively, and those of the original TransT algorithm are 90.9% and 91.7%, respectively. The precision of the KCF algorithm for both markers is the lowest, at 89.4% and 86.8%, respectively. However, in the closed-loop control of the PMSpM, the real-time performance of the visual attitude feedback is equally critical. The average processing speeds of the four algorithms under the same experimental platform are presented in Table 1.

In the visual feedback control loop, the phase lag caused by the computational latency will decrease the phase margin of the system and thereby affect the system stability [18]. Considering that the investigated PMSpM possesses fast dynamic response characteristics, the tracking algorithm should balance precision and processing speed as much as possible. As indicated by Table 1, an average latency of 16.33 ms and a frame rate of 61.23 FPS are achieved by the proposed GK-TransT while ensuring a high tracking precision, exhibiting better real-time performance compared with the original TransT.

Further analysis of Table 1 reveals that, due to its reliance on spatial regularization and iterative optimization solving, a single-frame processing latency of 67.62 ms is exhibited by the CSRT algorithm, which fails to meet the aforementioned real-time requirements. Meanwhile, two independent models are required to be run in parallel by the original TransT algorithm when processing the dual markers, resulting in an inference latency of 31.23 ms.

The proposed GK-TransT algorithm is based on a “single-model guidance and geometric assistance” architecture. The attention range is restricted by the Kalman filter, and the network computational overhead for the secondary marker is stripped away utilizing the rigid body prior. The test data demonstrate that a single-frame processing latency of 16.33 ms is achieved by the proposed GK-TransT, representing a reduction of 47.7% compared with the original dual-stream TransT scheme, with the average frame rate reaching 61.23 FPS. The above results indicate that the control requirements of the closed-loop system for a high frame rate and a low latency are effectively met by the proposed algorithm.

To further explain the reduction in processing latency, the computational complexity of the algorithm is analyzed in terms of model size, inference cost, and scalability.

1. Model Size: The proposed method does not expand the backbone architecture of the TransT network. Consequently, GK-TransT introduces no additional deep network parameters, and its model scale remains consistent with a single standard TransT model.
2. Inference Cost: Let $C_{T}$ denote the primary inference computational cost of a single TransT tracker, while $C_{K}$ and $C_{G}$ represent the additional overhead for Kalman prediction and geometric constraint calculation, respectively. Since the latter two are lightweight operations, the condition $C_{K},C_{G}\ll C_{T}$ is satisfied. For a dual-target tracking task, the original TransT requires parallel instantiation of two models, resulting in an inference cost of $2C_{T}$; in contrast, the total inference cost of GK-TransT is $C_{T}+C_{K}+C_{G}$. Therefore, the overall computational overhead is reduced by approximately 50%.
3. Scalability: When the visual tracking task is extended to N targets on the rotor surface, the computational complexity of the original TransT grows linearly with the number of targets, i.e., $O(N\cdot C_{T})$. In GK-TransT, the computational complexity of the deep network remains at a constant level of $O(1\cdot C_{T})$, while only the geometric calculation complexity expands to $O((N-1)\cdot C_{G})$. The analysis indicates that GK-TransT reduces the frequency of deep network invocations, thereby suppressing the growth of inference overhead as the number of tracking targets increases.

### 4.3. Verification of Algorithm Robustness

Since the CSRT and original TransT algorithms fail to meet the real-time standard of closed-loop control, the tracking robustness evaluation under complex working conditions was conducted only for the GK-TransT and the KCF algorithms in this study.

#### 4.3.1. Occlusion Robustness Comparison

In practical applications, environmental interferences such as target occlusion or sample contamination are frequently encountered by the visual attitude estimation system. To evaluate the dynamic tracking performance of the proposed GK-TransT algorithm under complex working conditions, a synthetic occlusion test case was constructed based on the offline visual datasets. Specifically, during frames 100 to 110 of the testing sequence, the bounding boxes of the primary and secondary markers are dynamically acquired through HSV color space threshold segmentation, and a pure black (zero grayscale value) solid rectangular occlusion block is synchronously superimposed within a region expanded by a 20-pixel margin outside them to achieve complete occlusion of the tracked targets. While the occlusion variable is introduced in this test scheme, the labeled physical ground truth of the feature points in the original video sequence is retained, whereby the occlusion resistance capabilities of different algorithms can be quantitatively evaluated.

**Figure 12:**
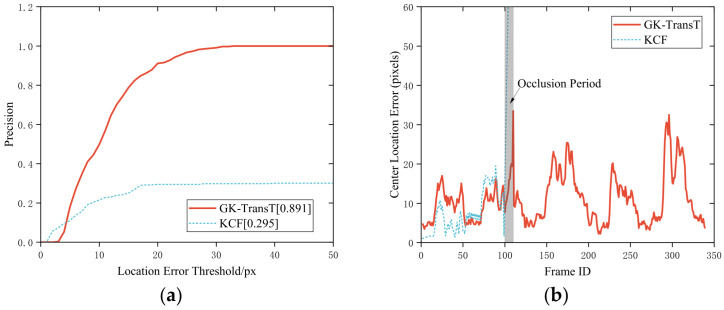

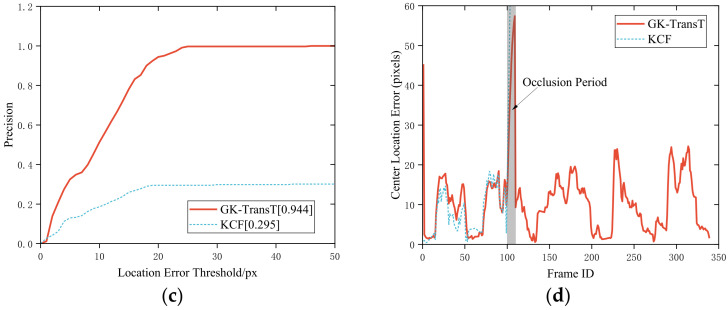
Algorithm tracking precision and center location error with occlusion: (**a**) Tracking precision of the primary marker. (**b**) Center location error curve of the primary marker. (**c**) Tracking precision of the secondary marker. (**d**) Center location error curve of the secondary marker.

**Figure 13:**
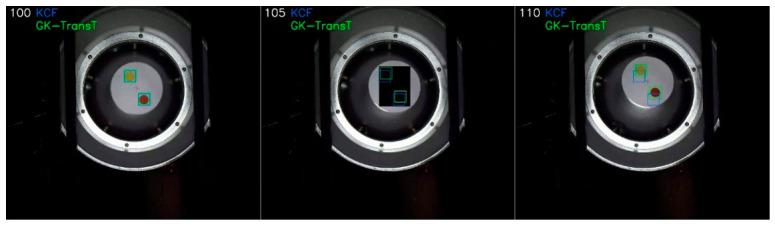
Tracking occlusion example.

The tracking precisions of the GK-TransT and the KCF algorithms under the aforementioned occlusion condition are compared in Figure 12. Quantitative analysis indicates that the tracking precisions of the GK-TransT algorithm for the main and auxiliary feature points are 0.891 and 0.944, respectively, whereas the precision of the KCF algorithm under the same conditions is 0.295. Further analysis of the error curves in Figure 12b,d reveals that target loss occurs for the KCF algorithm when the occlusion occurs at frame 100; its center location error diverges rapidly and exceeds the display range of the chart, and the tracking cannot be recovered. In contrast, valid position coordinate estimations are continuously output during the occlusion period by the proposed GK-TransT algorithm with the introduced temporal prediction mechanism, and a stable tracking state is maintained after the interference ends. The visual tracking example under this occlusion testing condition is illustrated in Figure 13.

#### 4.3.2. Blur Robustness Comparison

To evaluate the robustness of the proposed algorithm under dynamic working conditions, a synthetic test case featuring a sudden speed change accompanied by blur was constructed based on the original experimental videos, addressing the rapid displacement and motion blur phenomena caused by the high-speed maneuvers of the PMSpM. Specifically, frames 130 to 160 of the original video sequence are selected as the disturbance range. Within this range, the equivalent motion speed of the target is increased to 5 times the original speed through temporal downsampling technology (with the sampling interval set to K = 5) to simulate the step change in the rotor angular velocity. Furthermore, to reproduce the camera imaging blur effect caused by this sudden high-speed change, multiple consecutive image frames (M = 6) are linearly superimposed and fused. The difficulty of visual feature extraction is increased by this processing. This test aims to verify the capability of the proposed GK-TransT algorithm to maintain tracking stability under working conditions where rapid target displacement and feature blur occur.

**Figure 14:**
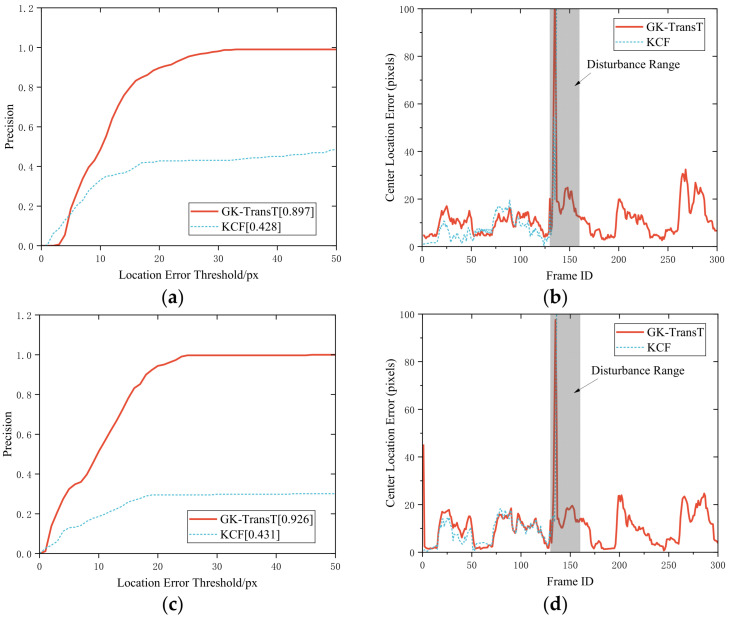
Algorithm tracking precision and center location error with blur: (**a**) Tracking precision of the primary marker. (**b**) Center location error curve of the primary marker. (**c**) Tracking precision of the secondary marker. (**d**) Center location error curve of the secondary marker.

**Figure 15:**
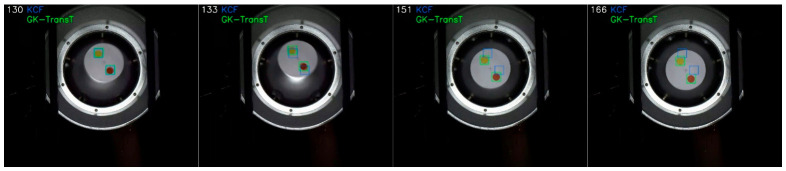
Tracking blur example.

The tracking precisions of the GK-TransT and KCF algorithms under the aforementioned blur condition are compared in Figure 14. Quantitative analysis indicates that the tracking precision rates of the GK-TransT algorithm for the primary and secondary markers are 0.897 and 0.926, respectively, whereas those of the KCF algorithm under the same conditions are 0.428 and 0.431. Further analysis of the center location error curves in Figure 14b,d reveals the target loss for the KCF algorithm when blur disturbance occurs around frame 135; its center location error diverges rapidly, exceeds the display range of the chart, and the tracking fails to recover. In contrast, during the blur disturbance period, the global attention mechanism of the Transformer network is utilized by the GK-TransT algorithm to overcome the influence of local feature degradation. The visual tracking example under this blur test condition is illustrated in Figure 15.

## 5. Verification of the GK-TransT-Based RAE System and the Sensor System

**Figure 16:**
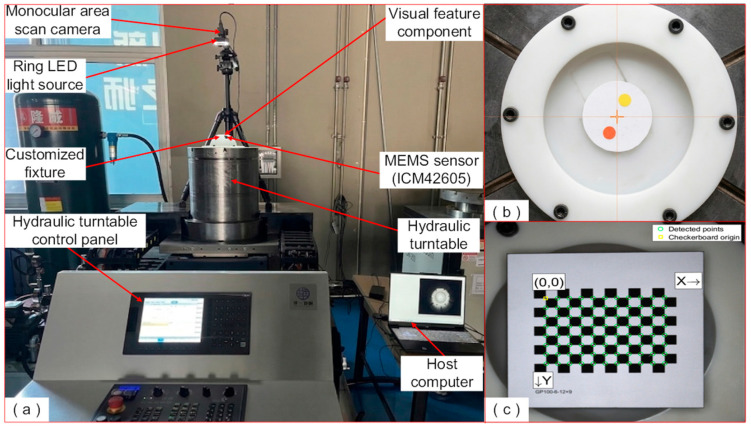
PMSpM RAE system verification platform: (**a**) RAE test bench; (**b**) VFC; (**c**) camera calibration.

To verify the effectiveness of the proposed GK-TransT-based RAE method, a MEMS inertial sensor is introduced as a control group, and the RAE test bench shown in Figure 16a is constructed. The hardware of this system mainly includes a customized fixture, a hydraulic rotary table, a Hikvision monocular area scan camera (Hikrobot, Hangzhou, China), a ring LED light source, an ICM42605 MEMS sensor (TDK InvenSense, San Jose, CA, USA), and a Python-based host computer system. Of this hardware, the customized fixture is mainly utilized to equivalently simulate the physical contour of the rotor bottom, serving as a rigid mounting base for the MEMS sensor. To provide a reliable attitude evaluation benchmark, the hydraulic rotary table adopted by the system possesses direct drive and closed hydrostatic support characteristics, whereby the motion hysteresis caused by mechanical friction can be avoided. Meanwhile, a circular grating ruler is built into the turntable, with an angular displacement measurement resolution of 0.2 arcseconds (0.2″), and its output data is utilized as the ground truth in the experiments. The key physical parameters of the laboratory setup are summarized in Table 2.

In the system layout, the VFC is fixed through the customized fixture (as shown in Figure 16b), and the monocular area scan camera is installed vertically directly above the VFC. To eliminate the interference of metal surface reflection on the calibration process, a high-precision alumina ceramic calibration checkerboard (Figure 16c) is adopted for the camera calibration. Furthermore, the VFC and the calibration checkerboard are maintained at the same height during installation to ensure a consistent object distance for calibration and measurement. When the relative installation position of the experimental device is changed, the on-site calibration is re-performed by the system to update the camera’s extrinsic parameters.

Based on the previously described pinhole camera model, let the feature point in the world coordinate system be denoted as $P_{w}={\left[X_{w},Y_{w},Z_{w},1\right]}^{T}$ and its corresponding image pixel coordinate be denoted as $p={\left[u,v,1\right]}^{T}$. The spatial mapping relationship between them can be formulated as

(25) $$S\left[\begin{array}{c} u\\ v\\ 1 \end{array}\right]=K\left[\begin{array}{cc} R & t \end{array}\right]\left[\begin{array}{c} X_{w}\\ Y_{w}\\ Z_{w}\\ 1 \end{array}\right],$$

where S denotes the scale factor; K represents the camera intrinsic matrix; and R and t denote the rotation matrix and translation vector describing the spatial pose of the camera (i.e., extrinsic parameters), respectively.

To uniformly evaluate the static tracking precision of the system under each degree of freedom, a standardized step response test protocol is formulated: the hydraulic rotary table is controlled to execute a single-axis motion at a predetermined step angle, and a step dwell period of 5 s is maintained after each step. In the data processing stage, the data segment within the step dwell period with a continuous 2.5 s (90 frames) angular fluctuation standard deviation of less than 0.3° is extracted by the system as the effective steady-state interval. To quantitatively evaluate the measurement precision, the Root Mean Square Error (RMSE) is introduced as a unified evaluation metric, and its calculation formula is

(26) $$RMSE=\sqrt{\frac{1}{N}\sum_{i=1}^{N}{\left(\theta_{\mathrm{measured}}(i)-\theta_{\mathrm{ref}}(i)\right)}^{2}},$$

where N is the total number of effective sampling frames extracted within the steady-state dwell period, $\theta_{\mathrm{measured}}(i)$ is the angle value obtained by the evaluated scheme (the GK-TransT visual algorithm or the MEMS sensor) at the i frame, and $\theta_{\mathrm{ref}}(i)$ is the corresponding physical reference ground truth of the hydraulic rotary table.

### 5.1. Spin Motion Comparison

In the spin motion test, the VFC is directly fixed on the testing reference surface of the hydraulic rotary table. The hydraulic rotary table is controlled to execute a single-axis step spin motion, where the testing range is set from 0° to 75° with a step angle of 10°, and a step dwell period of 5 s is maintained after each step. In the data processing stage, the steady-state data sequence within the 5 s step dwell period of the rotary table is directly extracted to calculate the static measurement error of the system.

**Figure 17:**
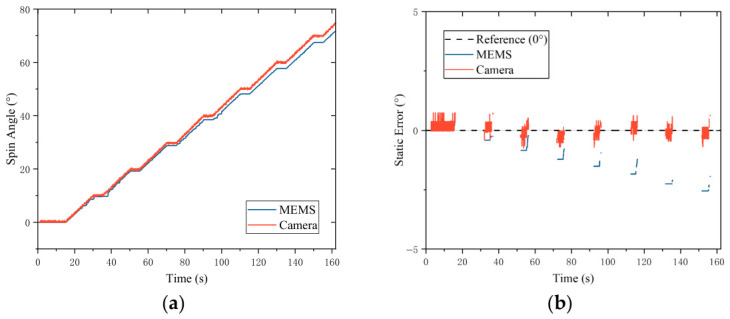
Spin angle steady-state error: (**a**) Spin angle trajectory. (**b**) Steady-state measurement error.

The experimental results are shown in Figure 17. Taking the physical angle during the step dwell period of the rotary table as the benchmark, the target pose can be stably tracked by the camera scheme. However, restricted by the inherent bias drift characteristics, obvious integral cumulative errors are generated in the measurement values of the MEMS sensor over time. The quantitative analysis indicates that the RMSE values of the camera scheme and the MEMS scheme are 0.2979° and 1.3120°, respectively. Based on the above results, it is concluded that higher measurement precision is achieved by the camera scheme compared with the MEMS scheme.

### 5.2. Tilt Motion Comparison

In the tilt motion test, the aforementioned customized fixture is utilized to simulate the tilt state of the rotor from 0° to 15°. During the testing process, the camera and the fixture are integrally rotated clockwise by 90°, while the MEMS sensor is maintained vertically. The rotor is controlled to execute a step tilt motion, where a step dwell period of 5 s is maintained after every 5° of tilt, whereby the steady-state interval data under the corresponding angles are extracted.

**Figure 18:**
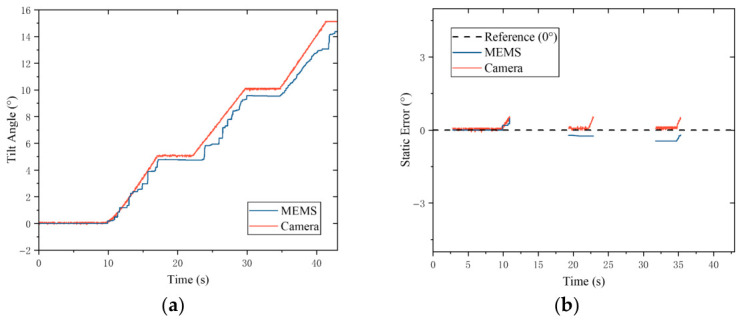
Tilt angle steady-state error: (**a**) Tilt angle trajectory. (**b**) Steady-state measurement error.

Similarly, taking the physical angle during the step dwell period of the rotary table as the benchmark, it is found from the comparative analysis of Figure 18 that smaller fluctuations relative to the reference waveform are exhibited by the tilt angle detection results of the camera scheme compared with the MEMS scheme. Quantitative evaluation revealed that the RMSE values of the camera scheme and the MEMS scheme are 0.1496° and 0.2640°, respectively. Based on these results, the conclusion that higher measurement precision is achieved by the camera scheme compared with the MEMS scheme in the RAE system is further proved.

### 5.3. Yaw Motion Comparison

The comparative test of the yaw motion is conducted on the aforementioned experimental platform. First, the rotor is fixed at an initial tilt state deflected by 15° around the Y-axis utilizing the customized fixture; subsequently, the hydraulic rotary table is controlled to execute the yaw motion around the global Z-axis. The motion strategy is set to a step angle of 10°, and a step dwell period of 5 s is maintained after each step, during which the attitude data of the rotor are synchronously collected.

Under this working condition, due to the existence of the initial tilt angle of the rotor, a conical motion trajectory is formed when the global yaw motion is executed by the rotor. Although the direct object detected by the camera scheme is the global absolute yaw attitude around the Z-axis, the local coordinate axes of the MEMS sensor fixed on the rotor are constantly deflected relative to the gravity coordinate system. At this time, deep coupling occurs in the Euler angles output by the MEMS sensor, and direct comparison with the visual method cannot be performed by simply locking a single degree of freedom. To solve this problem, an attitude decoupling method based on the $SO\left(3\right)$ rotation matrix is adopted in this article. First, the coupled Euler angles $\left(\psi_{m},\theta_{m},\phi_{m}\right)$ output by the MEMS are converted into the global rotation matrix $R_{b}^{n}$:

(27) $$R_{b}^{n}=R_{z}(\psi_{m})R_{y}(\theta_{m})R_{x}(\phi_{m})=\left[\begin{array}{ccc} R_{11} & R_{12} & R_{13}\\ R_{21} & R_{22} & R_{23}\\ R_{31} & R_{32} & R_{33} \end{array}\right].$$

Subsequently, the column vector $x_{n}={\left[R_{11},R_{21},R_{31}\right]}^{T}$ characterizing the local X-axis direction of the sensor in the rotation matrix is extracted. After it is projected onto the global horizontal plane, the decoupled global absolute yaw angle $\Psi_{\mathrm{global}}$ is calculated utilizing the four-quadrant arctangent function:

(28) $$\Psi_{\mathrm{global}}=\arctan(R_{21},R_{11}).$$

**Figure 19:**
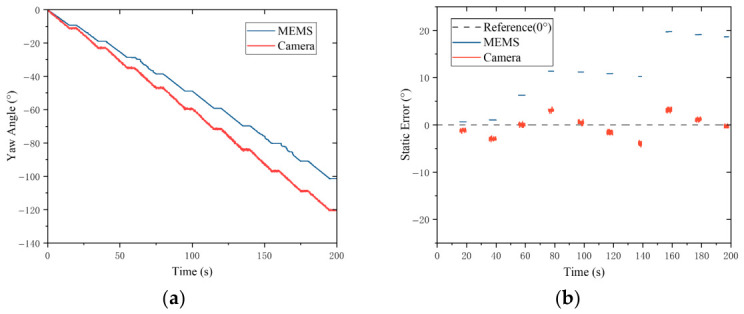
Yaw angle steady-state error: (**a**) Yaw angle trajectory. (**b**) Steady-state measurement error.

After unifying the yaw benchmark using the above decoupling algorithm, temporal phase unwrapping is processed by the host computer program on the absolute yaw angles calculated by both the visual and the MEMS schemes to eliminate the non-physical mathematical jumps of the attitude angles at the boundaries of 0 and 2π. The initial bias zeroing is also executed, thereby allowing the continuous and cumulative physical motion trajectory of the rotor to be reconstructed. The comparative results are shown in Figure 19.

According to the comparative analysis in Figure 19, higher stability is exhibited by the camera scheme during the attitude tracking process, and its measurement deviation is smaller than that of the MEMS scheme. The superiority of this scheme is further confirmed by the quantitative analysis results: the RMSE of the camera scheme is 0.8847°, and that of the MEMS scheme is 15.0819°.

Based on the comprehensive analysis of the above experimental results, it is concluded that stable 3-DOF attitude measurement is achieved by the proposed GK-TransT-based camera scheme, and the long-term cumulative error inherent in traditional inertial sensors is avoided. It can serve as an effective attitude recognition method for the closed-loop control of the PMSpM.

## 6. Conclusions

This article proposes a GK-TransT-based visual RAE method for the PMSpM. First, the PMSpM is adopted as the research object, and its basic structure is introduced. The calculation method of the Euler angles is also described. Subsequently, the GK-TransT algorithm for the RAE system is proposed, and the precision, processing speed, and robustness of the proposed algorithm are verified through a single-target tracking comparison among the CSRT, KCF, and original TransT algorithms. Specifically, the experimental results show that the proposed GK-TransT algorithm achieves tracking precisions of 90.9% and 94.4% for the main and auxiliary feature points, respectively, with an average processing speed of 61.23 FPS and a single-frame latency of 16.33 ms. Furthermore, to verify the accuracy of the proposed GK-TransT-based RAE system, an RAE test bench was developed to compare the measurement results between the camera scheme and MEMS scheme, where the static result of the hydraulic rotary table is set as the physical benchmark. The experimental results indicate that the proposed GK-TransT algorithm is highly applicable to the RAE system, and higher tracking precision is achieved compared with the MEMS inertial sensor. In summary, the effectiveness of the proposed visual RAE system is verified, which can be utilized to support the subsequent closed-loop motion control and system performance improvement for the PMSpM.

Beyond the specific application to the PMSpM, the proposed method can be further extended to applications such as multi-degree-of-freedom robotic joints, industrial gimbals, and visual servo systems with short-term occlusion or visual interference. Since the proposed approach integrates visual tracking, Kalman-based temporal prediction, and rigid-body geometric constraints, its fundamental framework can also provide a reference for attitude or pose estimation in dynamic systems with observable visual features and rigid-body motion relationships. By enhancing the continuity and robustness of visual feature tracking under complex conditions, this method is expected to improve the state feedback accuracy in related dynamic control systems.

## Figures and Tables

**Table 1:** Processing speeds of the tracking algorithms.

Algorithm	FPS	Latency (ms)
GK-TransT	61.23	16.33
Original TransT	32.02	31.23
KCF	92.05	10.86
CSRT	14.79	67.62

**Table 2:** Key physical parameters of the laboratory setup.

Component	Parameter	Value
PMSpM rotor	Physical radius (*R*)	65 mm
Monocular camera	Model	Hikvision MV-CA050-12UC
Monocular camera	Resolution	2448 × 2048 pixels
MEMS sensor	IMU chip model	ICM42605
MEMS sensor	Gyroscope measurement range	±2000°/s
MEMS sensor	Accelerometer measurement range	±16 g
MEMS sensor	Sampling rate	200 Hz
Hydraulic turntable	Measurement resolution	0.2 arcsec

## Data Availability

The data and materials in this article will be made available upon reasonable request.
